# Activity of Ethanolic and Supercritical Propolis Extracts in *Corynebacterium pseudotuberculosis* and Its Associated Biofilm

**DOI:** 10.3389/fvets.2021.700030

**Published:** 2021-09-01

**Authors:** Laerte Marlon Santos, Daniela Méria Rodrigues, Maurício Alcantara Kalil, Vasco Azevedo, Roberto Meyer, Marcelo Andres Umsza-Guez, Bruna Aparecida Machado, Nubia Seyffert, Ricardo Wagner Portela

**Affiliations:** ^1^Laboratório de Imunologia e Biologia Molecular, Instituto de Ciências da Saúde, Universidade Federal da Bahia, Salvador, Brazil; ^2^Instituto de Tecnologia em Saúde, CIMATEC-SENAI, Salvador, Brazil; ^3^Laboratório de Genética Celular e Molecular, Instituto de Ciências Biológicas, Universidade Federal de Minas Gerais, Belo Horizonte, Brazil; ^4^Instituto de Ciências da Saúde, Universidade Federal da Bahia, Salvador, Brazil

**Keywords:** caseous lymphadenitis, small ruminants, microbial resistance, natural extracts, supercritical extraction

## Abstract

*Corynebacterium pseudotuberculosis* is the etiological agent of caseous lymphadenitis in small ruminants, a chronic disease characterized by the development of granulomas in superficial and visceral lymph nodes as well as in several organs. An important characteristic of the infection with this bacterium is the formation of a biofilm and the absence of effective antibiotic therapy against the disease. From this scenario, the objective of this study was to evaluate the susceptibility of *C. pseudotuberculosis* to conventional antibiotics and to red, green, and brown propolis extracts obtained by the supercritical and ethanolic extraction methods as well as its activity in the bacterial biofilm. The results of the sensitivity test using antibiotics indicated a sensitivity of *C. pseudotuberculosis* strains to the antimicrobial agents. The ethanolic extract of green propolis and the supercritical red propolis extract showed the best antibacterial activities against planktonic *C. pseudotuberculosis*. A lower antimicrobial activity of the brown propolis extract was identified. Propolis extracts were effective in interfering with the formation of the *C. pseudotuberculosis* biofilm but had little activity on the consolidated biofilm. In conclusion, propolis extracts are more effective against *C. pseudotuberculosis* in the planktonic stage, being able to interfere with the formation of bacterial biofilm. However, the action of propolis extracts in a sessile and structured microbial biofilm is reduced.

## Introduction

*Corynebacterium pseudotuberculosis* is a Gram-positive and intracellular bacterium that causes caseous lymphadenitis (CLA) in small ruminants, a chronic disease characterized by the development of granulomas in superficial and visceral lymph nodes as well as in several organs. CLA causes weight loss, reduced production of milk, and a decrease of the commercial value of the leather, characterizing its economic importance (1, 2). Antibiotic therapy is **one** of the strategies used to control CLA, but the treatment is long term with high costs and low efficacy. Even though it is a bacterium sensitive to most antibiotics, the therapy becomes inefficient when the bacterial biofilm is formed (3) and in encapsulated abscesses (4). In this way, the current treatment of the disease is based on opening, draining, and cleaning the lesions, which leads to environmental contamination and recurrences (5).

Propolis, a low-cost natural resinous mixture produced by honeybees from substances collected from parts of plants, buds, and exudates, is seen as an alternative and complementary treatment against several infectious diseases, especially in cases in which resistance to clinically relevant antimicrobials is already widespread and conventional treatments are not effective. The antimicrobial, wound-healing, anti-inflammatory, and immunomodulatory activities of propolis extracts are attributed to many compounds present in its composition, mainly flavonoids, phenolic acids, and their esters (6). Due to the constant development of resistance to drugs by bacteria, propolis can be a feasible treatment option because its biological activity is a consequence of the synergism of various chemical compounds present in the extract, and the combination with antimicrobial agents may allow lower doses of antibiotics (7). Propolis can reduce biofilm formation and accelerate healing processes (8), especially in cases of chronic infections that are difficult to control due to biofilm formation in the wound environment (9). This situation was evident in clinical isolates of *Proteus mirabilis* from patients with chronic wounds, for which ethanolic extracts of propolis were effective in inhibiting the formation of biofilm (10).

Due to the lack of efficient treatment against CLA, its dissemination among small ruminant herds around the world has generated significant economic losses (11, 12) and the need for products that can prevent the infection by the agent and its spread in the environment and in the herds; new alternatives therapies are necessary for the treatment and/or prevention of the disease (13). Therefore, the present study aimed to evaluate the antibacterial and antibiofilm effects of different types of propolis extracts on *C. pseudotuberculosis* strains and its associated biofilm.

## Materials and Methods

### Obtaining, Processing, and Characterization of the Propolis Samples

The red propolis (RAL) samples originated from the state of Alagoas, Brazil, and the samples of brown (BSC) and green (GPR) propolis were obtained in the states of Santa Catarina and Paraná, respectively. The propolis samples were ground in a grinder and then sieved (60 mesh) to obtain adequate granulometry (~0.250 mm), thus increasing the surface area, and homogenized. The contents of moisture, protein, ash, total lipids, minerals, and quantification of water activity of red, green, and brown propolis extracts used in this study are described by Machado et al. (14) and are listed in Supplementary Material 1.

### Obtaining of the Ethanolic and Supercritical Propolis Extracts

The ethanolic extracts of propolis were obtained according to the protocol described by Machado et al. (14); 15 mL of ethanol 80% were added to 2 g of propolis. The extraction was conducted in a temperature of 70°C for 30 min under constant agitation in a shaker incubator. Then, the extract was centrifuged at 6,000 *x g* for 11 min at 5°C. At the end of the centrifugation, the supernatant was transferred to a 50-ml tube, 10 ml of ethanol 80% were added to the residue, and centrifugation was repeated. The supernatants were homogenized and maintained at 50°C until completely dry. Then, the extracts were stored in tubes covered with aluminum foil under inert atmospheric conditions (N_2_) to avoid degradation of the material.

The supercritical extracts of propolis were obtained according to Machado et al. (14). The equipment used to obtain the propolis extracts was a pilot unit called SFT-110 Supercritical Fluid Extractor (Supercritical Fluid Technologies Inc., Newark, DE, USA), composed of a high-pressure pump (capacity up to 10,000 psi), extraction cell (capacity 100 mL), oven (containing a preheater), static/dynamic and restrictor valves, flow meter, and a flow totalizer. A CO_2_ cylinder with a fishing tube was used to ensure that only liquid CO_2_ was used in the system. CO_2_ was not reused in the system. The extraction cell consisted of a package with 7.5 g of propolis sample homogenized with 1% ethanol (w/w) as co-solvent, wool and glass beads to avoid preferential paths of CO_2_ and total filling of the bed. The temperature of the restrictor valve was set at 80°C for all extraction processes. The extracts were collected in 50-mL glass flasks immersed in ice at room pressure. The flasks containing the extracts were protected with aluminum foil under inert atmospheric conditions (N_2_) to avoid material degradation and kept at 4°C until analysis. The chemical composition of the propolis extracts can be found at the Supplementary Material 2.

### Bacterial Strains

Four strains of *C. pseudotuberculosis* were used in this study: the 1002 strain (15–18), which is the standard strain used for the bacterial genome sequencing project; the VD57 strain, a highly pathogenic isolate (16, 19, 20); the viscerotropic N1 strain (21); and the CAPJ4 strain, which is a biofilm-producing strain (22–24). Some characteristics of these strains are listed in Table 1.

**Table 1:** Characteristics of the *Corynebacterium pseudotuberculosis* strains used in this study.

**Strain**	**Origin**	**History**	**Pathogenicity**	**GenBank**	**References**
1002	Curaça county, Bahia State, Brazil, 1971	Isolated from a goat with caseous lymphadenitis	Strain used as a reference for the *C. pseudotuberculosis* genome sequencing project	*CP001809.2*	(15–18)
VD57	Municipality of Juazeiro, Bahia state, Brazil, 2008	Isolated from a granulomatous lesion of a goat	Highly pathogenic to goats and mice; induce IFN-gamma production in goats and a high mortality in mice	*CP009927.1*	(16, 19, 20)
N1	Mongomo region, Equatorial Guinea, 2016	Isolated from the lung of a sheep	Viscerotropic strain	*CP013146*	(21)
CAPJ4	Municipality of Juazeiro, Bahia state, Brazil, 2013.	Isolated from a granulomatous lesion of a goat	Biofilm-forming strain; colonize several host tissues	*CP026499*	(22–24)

*Listed is the geographical origin of the strain, host, virulence profile, and the ID of the complete genome sequence at the GenBank*.

### Determination of *C. pseudotuberculosis* Sensitivity to Clinically Relevant Antimicrobials Using Disk Diffusion Methodology

The evaluation of the susceptibility of 1002, VD57, and N1 strains to clinically relevant antibiotics was initially carried out using disk diffusion methodology (25) with some modifications. The strains were grown in Brain-Heart Infusion (BHI, Himedia, Mumbai, India) 0.1% Tween 80 at 37°C for 24 h. Afterward, the turbidity of the culture in BHI broth was adjusted to obtain an optical turbidity comparable to that of the 0.5 McFarland standard solution. This resulted in a suspension containing ~2 x 10^8^ CFU/mL.

Briefly, the bacterial inoculum was seeded on a BHI agar (Himedia, Mumbai, India) surface in different directions, covering the entire surface of the plate. After 5 min, disk impregnated with antibiotics (sulfazotrin 25 μg, amikacin 30 μg, ampicillin 2 μg, cefoxitin 30 μg, doxycycline 30 μg, ciprofloxacin 5 μg, clindamycin 2 μg, penicillin 10 U, amoxicillin 10 μg, erythromycin 15 μg, cefotaxime 30 μg, enrofloxacin 5 μg, oxacillin 1 μg, tetracycline 30 μg, bacitracin 10 μg, norfloxacin 10 μg, gentamicin 10 μg, and cephalothin 30 μg) were added with the aid of a sterile forceps. The plates were then incubated for 48 h at 37°C. The inhibition halos were measured and compared to pre-established standards (25) for determination of *C. pseudotuberculosis* susceptibility to antibiotics (Table 2). Three independent assays were performed.

**Table 2:** Antimicrobial sensitivity profile of *C. pseudotuberculosi* as determined by the disk diffusion method.

**Antibiotic**	***C. pseudotuberculosis*** **strain**		
	**1002**	**VD57**	**N1**
Sulfazotrin (25 μg)	S	S	S
Amikacin (30 μg)	S	S	S
Ampicillin (2 μg)	S	S	S
Cefoxitin (30 μg)	S	S	S
Doxycycline (30 μg)	S	S	S
Ciprofloxacin (5 μg)	S	S	S
Clindamycin (2 μg)	S	S	S
Penicillin (10 U)	S	S	S
Amoxicillin (10 μg)	S	S	S
Erythromycin (15 μg)	S	S	S
Cefotaxime (30 μg)	S	S	S
Enrofloxacin (5 μg)	S	S	S
Oxacillin (1 μg)	I	R	R
Tetracycline (30 μg)	S	S	S
Bacitracin (10 μg)	S	S	S
Norfloxacin (10 μg)	S	S	S
Gentamicin (10 μg)	S	S	S
Cephalotin (30 μg)	S	S	S

*The classification of the strain as resistant, intermediate, or sensitive was made according to the CLSI (12). S, sensitive; I, Intermediate; R, resistant*.

*The results correspond to three independent experiments*.

### Determination of Clinically Relevant Antimicrobials and Propolis Extracts Minimum Inhibitory Concentration (MIC) and Minimum Bactericidal Concentration (MBC)

Phosphate buffer solution (pH 6.0; 0.1 mol/L) and ultrapure water were used as diluents for the preparation of standard solutions of antibiotics as described by the Clinical & Laboratory Standards Institute (CLSI) (26), and the propolis extracts were diluted in BHI broth supplemented with Tween 80 1%. BHI broth supplemented with 0.5% Tween 80 (Sigma Aldrich, Saint Louis, MI) was used as the first-choice medium for sensitivity tests for *C. pseudotuberculosis*. The MIC methodology was carried out according to Norman et al. (27) with some modifications. After incubation, each strain was diluted in BHI broth to achieve an optical density of 0.08–0.10 at a wavelength of 600 nm (optically comparable to the 0.5 McFarland standard solution). Each suspension contained ~2 x 10^6^ CFU/mL of *C. pseudotuberculosis*. Then, the suspensions were immediately diluted in BHI broth to obtain a concentration of 1 x 10^6^ CFU/mL. To reach a final concentration of 5 x 10^5^ CFU/mL in the wells of the culture plate, each well was inoculated with 100 μL of the inoculum and 100 μL of solutions containing the antibiotic or propolis extracts in different concentrations. The plates were incubated at 37°C for 48 h. The analysis was performed on a spectrophotometer (Bio-Rad, Hercules, CA, USA) at a wavelength of 600 nm, and the minimum concentration capable of totally inhibiting bacterial growth (MIC_100_) was established.

A 20-μL aliquot was removed from the microdilution assay wells and inoculated on BHI agar plates. The plates were incubated at 37°C and, after 48 h, the lowest concentration of the antimicrobial agent capable of completely causing bacterial death (MBC_100_) was defined. Three different controls were used: a positive control, composed of the bacterial inoculum and BHI broth; a negative control, composed of BHI broth and the different antibiotic and propolis dilutions without bacterial inoculum; and a second negative control, composed of BHI broth only. The following antibiotics were used (Table 3): amoxicillin (0.048–25 μg/mL), cephalothin (0.234–120 μg/mL), ceftriaxone (0.390–200 μg/mL), clindamycin (0.048–25 μg/mL), doxycycline (0.097–50 μg/mL) and penicillin (0.0005–0.134 μg/mL). Concentrations of propolis extracts ranged from 0.016 to 8 mg/mL. Three independent assays were performed.

**Table 3:** MIC_100_ and MBC_100_ of six antimicrobial agents against *C. pseudotuberculosis* isolates as determined by the broth microdilution method.

		***C. pseudotuberculosis*** **strains**					
		**1002**		**VD57**		**N1**	
**Antibiotic**	**Concentration range (μg/mL)**	**MIC_**100**_**	**MBC_**100**_**	**MIC_**100**_**	**MBC_**100**_**	**MIC_**100**_**	**MBC_**100**_**
Amoxicillin	0.048–25	1.56	1.56	3.13	6.25	1.56	1.56
Cephalotin	0.234–120	0.94	0.94	0.94	0.94	0.94	0.94
Ceftriaxone	0.390–200	0.39	0.39	0.78	1.56	0.78	0.78
Clindamycin	0.048–25	0.09	0.09	0.39	0.39	0.39	0.78
Doxycycline	0.097–50	6.25	6.25	0.39	0.39	12.5	12.5
Penicillin	0.0005–0.134	0.001	0.002	0.001	0.001	0.001	0.0005

*MIC_100_ and MBC_100_ results are expressed as means of three independent assays in mg/mL*.

### Biofilm Production Assay

The semiquantitative analysis of biofilm production followed the methodology described by Kalil et al. (2). *C. pseudotuberculosis* were inoculated in 3 mL of Tryptone Soy Broth (TSB, Himedia, Mumbai, India) and incubated at 37°C until obtaining an optical density (OD) of 0.2 at a wavelength of 600 nm. Then, 200 μL of this bacterial suspension was transferred to sterile untreated polystyrene microplates and incubated at 37°C for 48 h. After incubation, the contents of each well were aspirated, and the wells were washed twice with 200 μL of PBS pH 7.2. The biofilm was then fixed with 200 μL of methanol and left in the incubator until drying. The wells were then stained for 5 min with 200 μL of a 2% crystal violet solution and then washed with distilled water. The dye impregnated in the biofilm was then eluted with 160 μL of a 33% acetic acid solution. As a negative control for this test, we used wells with TSB and without inoculum. The OD of each well was measured at a wavelength of 595 nm. Three independent assays were performed.

To characterize the intensity of biofilm formation, the following equations were used, in which ODI indicates the optical density of the isolate and ODNC represents the optical density of the negative control: ODI ≤ ODNC = no biofilm development; ODI / ODNC ≤ 2 = weak biofilm formation; ODI/ODNC ≤ 4 = moderate biofilm production capacity; ODI / ODNC> 4 = strong biofilm production capacity (28). The *C. pseudotuberculosis* CAPJ4 strain, a biofilm-producing strain, was added to the biofilm experiments as a positive control.

### Minimum Biofilm Inhibitory Concentration (MBIC) as Interference Assay in Consolidated Biofilm

The choice of propolis extracts for MBIC determination was based on the results of the antibacterial activity of ethanolic and supercritical extracts of red, green, and brown propolis against strains of *C. pseudotuberculosis* at the microdilution assay. The propolis extracts were diluted in TSB supplemented with Tween 80 1%. The assay for the determination of the antibiofilm activity of the propolis extracts followed the methodology described by Nostro et al. (28) with adaptations. *C. pseudotuberculosis* isolates were inoculated in 3 mL of TSB and incubated at 37°C for 48 h. After this period, the bacterial suspensions were standardized in TSB broth for an OD of 0.2 at a wavelength of 600 nm, and 200 μL of these suspensions were transferred to microplate wells and incubated at 37°C for 48 h. After consolidation of the biofilm, considering the results previously obtained for the MIC_100_ and MBC_100_ determination, 200 uL of the propolis extracts in different concentrations (8, 4, 2, and 1 mg/mL) were inoculated in the wells with the consolidated biofilm. Then, the microplates were incubated in a bacteriological incubator at 37°C for 48 h. The OD, measured at 595 nm, was determined immediately after the addition of the antimicrobial (DO 0 h) at 24 h (DO 24 h) and 48 h (DO 48 h) after the experiment. MBIC was defined as the lowest antimicrobial concentration in which there was no time-dependent increase in the OD when compared with the later exposure time (29). Three different controls were made: a negative control, composed of the biofilm and TSB only; a control composed of TSB broth and each propolis dilution; and a third control, made only with TSB broth.

### Biofilm Formation Interference Assay

To evaluate the action of propolis extracts on biofilm formation, *C. pseudotuberculosis* isolates were inoculated in 3 mL of TSB and incubated at 37°C for 48 h. After this period, the bacterial suspensions were standardized in TSB broth with an OD of 0.2 at a wavelength of 595 nm. Then, 100 μL of the standardized bacterial suspensions and 100 μL of propolis extracts in different concentrations (8, 4, 2, and 1 mg/mL) were inoculated into the wells of a sterile 96-well microplate and incubated at 37°C for 48 h. The formed biofilm was detected after 48 h, and the percentage of inhibition of biofilm production was calculated considering the bacterial control suspensions that were not incubated with extracts. Biofilm formation inhibition was defined as (OD595 from the treated well/OD595 from the control well) x 100 (2). Three different controls were made as previously described. Three independent assays were performed.

The percentage of biofilm formation inhibition and of consolidate biofilm disruption were calculated using the following formula (30):

$$\%\text{ inhibition=1 - }\frac{\text{OD595 of the treated }C.\;pseudotuberculosis}{\text{OD595 of the non - treated }C.\;pseudotuberculosis}\text{ x 100}$$

### Scanning Electron Microscopy (SEM)

For the SEM analysis, the bacterial biofilms (treated and untreated) were obtained as previously described with a modification represented by the addition of an untreated and sterile glass coverslip at the bottom of each well of an untreated 24-well culture plate, and these coverslips containing the treated and untreated biofilms were analyzed at the scanning electron microscope. The consolidated biofilms exposed or not to the propolis extracts were sequentially fixed in two different solutions: (i) 2.5% glutaraldehyde in 0.1 M sodium cacodylate buffer pH 7.4 for 2 h, followed by three washes with the same buffer and (ii) 1% osmium tetroxide in 0.1 M sodium cacodylate for 1 h at room temperature and washed three times with 0.1 M sodium cacodylate buffer, pH 7.4. After fixation, the biofilms were dehydrated in increasing concentrations of ethanol (30, 50, 70, 90%, and absolute alcohol) and dried with the CPD 030 Critical Point equipment (Leica, Wetzlar, Germany) with CO_2_ as a transition medium. The biofilms were examined using the SEM JSM-6390LV (Jeol, Tokyo, Japan) operated at 15 kV.

### Statistical Analysis

The results obtained for the inhibition of bacterial growth, and biofilm formation by different antimicrobials were submitted to the D'Agostino–Pearson test with the objective to assess its distribution. The one-way ANOVA and the *t-*test were used to compare the inhibition of the bacterial growth and biofilm formation interference by the different concentrations the propolis extracts. A value of *p* < 0.05 was considered significant. The statistical analyses described herein were conducted using the software SPSS v. 22.0 (IBM, Amonk, NY).

## Results

### Susceptibility of Planktonic *C. pseudotuberculosis* Strains to Antibiotics

The results of the disk diffusion assay indicated a high sensitivity of *C. pseudotuberculosis* strains to the antimicrobial agents. *C. pseudotuberculosis* strains VD57 and N1 showed resistance only to oxacillin (Table 2). When using the broth microdilution methodology with clinically relevant antimicrobials, the lowest MIC_100_ values were found for penicillin, cephalothin, ceftriaxone, and clindamycin. *C. pseudotuberculosis* was less sensitive to doxycycline with MIC intervals between 0.39 and 12.5 μg/mL (Table 3).

### Susceptibility of Planktonic *C. pseudotuberculosis* Strains to Propolis Extracts

*C. pseudotuberculosis* showed sensitivity to all tested propolis extracts (Table 4). The strains tested against the supercritical extract of red propolis showed values of MIC_100_ and MBC_100_ equal to 4 mg/mL except for strain 1002, which presented an MBC_100_ value equal to 8 mg/mL. The ethanolic extract of green propolis showed the lowest MIC_100_ value among the tested extracts (2 mg/mL for strain 1002). A lesser antimicrobial activity of brown propolis extracts was observed, and we found MIC_100_ and MBC_100_ values equal to or >8 mg/mL among the tested isolates. However, this extract completely inhibited the bacterial growth of the VD57 strain at MIC_100_ and MBC_100_ equal to 8 mg/mL.

**Table 4:** MIC_100_ and MBC_100_ of supercritical and ethanolic extracts of red, green, and brown propolis against isolates of *C. pseudotuberculosis* as determined by the broth microdilution method.

		***C. pseudotuberculosis*** **strain**					
		**1002**		**VD57**		**N1**	
**Propolis extract**	**Concentration range (ug/mL)**	**MIC_**100**_**	**MBC_**100**_**	**MIC_**100**_**	**MBC_**100**_**	**MIC_**100**_**	**MBC_**100**_**
RAL SCO2	0.016–8	4	8	4	4	4	4
RAL EtOH	0.016–8	4	8	8	8	4	4
GPR SCO2	0.016–8	4	8	>8	>8	4	8
GPR EtOH	0.016–8	2	4	8	8	4	4
BSC SCO2	0.016–8	>8	>8	>8	>8	>8	>8
BSC EtOH	0.016–8	>8	>8	8	8	>8	>8

*MIC_100_ and MBC_100_ results are expressed in mg/ml and represent the means of three independent assays. RAL, red propolis from Alagoas State; GRP, green propolis from Paraná State; BSC, brown propolis from Santa Catarina State; SCO2, supercritical extract; EtOH, ethanolic extract*.

### Production of Biofilm by *C. pseudotuberculosis* Strains and Interference of Biofilm Production and of the Consolidated Biofilm by Propolis Extracts

**Figure 1:**
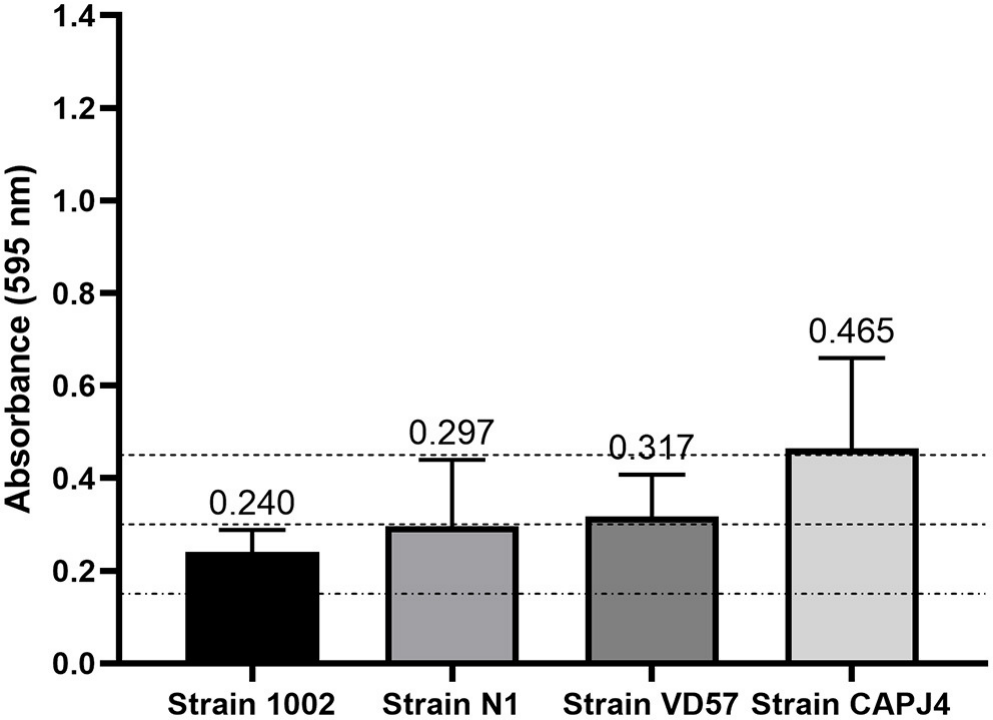
Semi-quantification of biofilm production by *C. pseudotuberculosis* reference strains. The dashed lines indicate the mean value of the negative control (DO595 <0.150), weak biofilm production (DO595 between 0.150 and 0.300), moderate biofilm production (DO595 between 0.300 and 0.450), and strong biofilm production (DO595 > 0.450). The bars indicate the standard deviation of three independent experiments, each one made with three replicates.

The biofilm production assay classified all bacteria as biofilm-forming isolates. Strains 1002 and N1 were characterized as low producers of biofilm. The VD57 strain presented a moderate biofilm production. The CAPJ4 strain was characterized as a strong biofilm producer (Figure 1).

**Figure 2:**
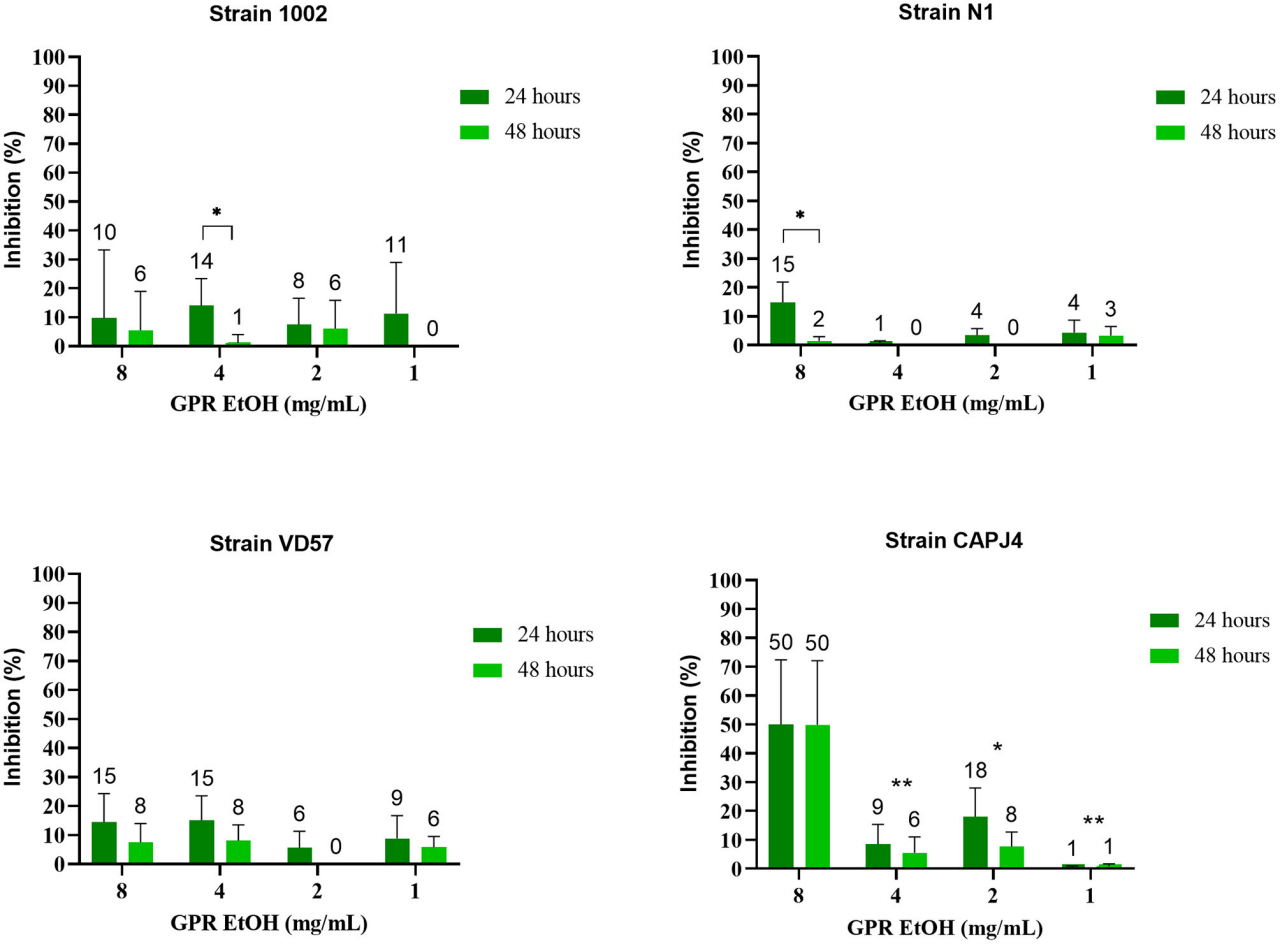
Activity of the green propolis ethanolic extract (GPR EtOH) in the *C. pseudotuberculosis* consolidated biofilm. The bars represent the standard deviation of three independent experiments. The values above the bars represent the average value of consolidated biofilm disruption (%). The asterisks indicate statistical differences between the bacterial cells treated with different concentrations of propolis extracts at different times, analyzed by one-way ANOVA and *t*-test. *p = 0.02; **p < 0.006.

**Figure 3:**
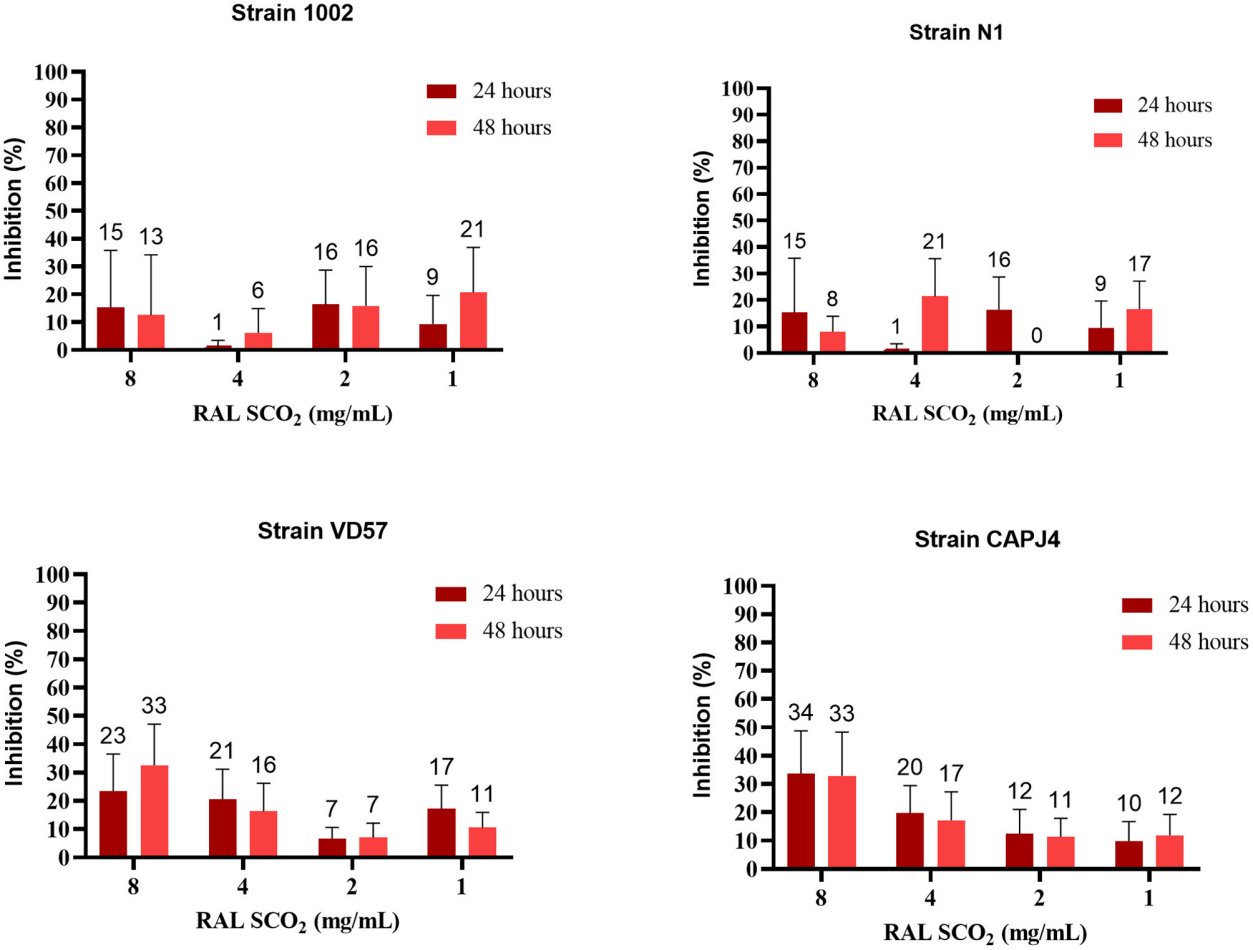
Antibiofilm activity of the supercritical extract of red propolis (RAL SCO_2_) in the consolidated biofilm of *C. pseudotuberculosis*: Strain 1002, Strain N1, Strain VD57, Strain CAPJ4. The bars represent the standard deviation of three different experiments. The values above the bars represent the average value of consolidated biofilm disruption (%). The asterisks indicate statistical differences between the bacterial cells treated with different concentrations of propolis extracts and at different times, analyzed by the one-way ANOVA and *t-*tests. No significant differences were found (*p* < 0.05) when comparing different concentrations of propolis extracts in different incubation periods.

The ethanolic extract of green propolis demonstrated low efficacy in disrupting the consolidated biofilm of *C. pseudotuberculosis*. Strains 1002, N1 and VD57 showed sensitivity equal to or <15% at the highest propolis extract concentrations tested herein. The biofilm producer CAPJ4 strain showed an inhibition value equal to 50% at a concentration of 8 mg/mL after 48 h of incubation (Figure 2). Regarding red propolis supercritical extract inhibition of the consolidated biofilm (Figure 3), it was evident that the activity on *C. pseudotuberculosis* CAPJ4 strain biofilm was reduced when compared with the ethanolic extract of green propolis. The percentages of inhibition of the strains VD57 and CAPJ4 reached a maximum of 33% after 48 h of incubation.

**Figure 4:**
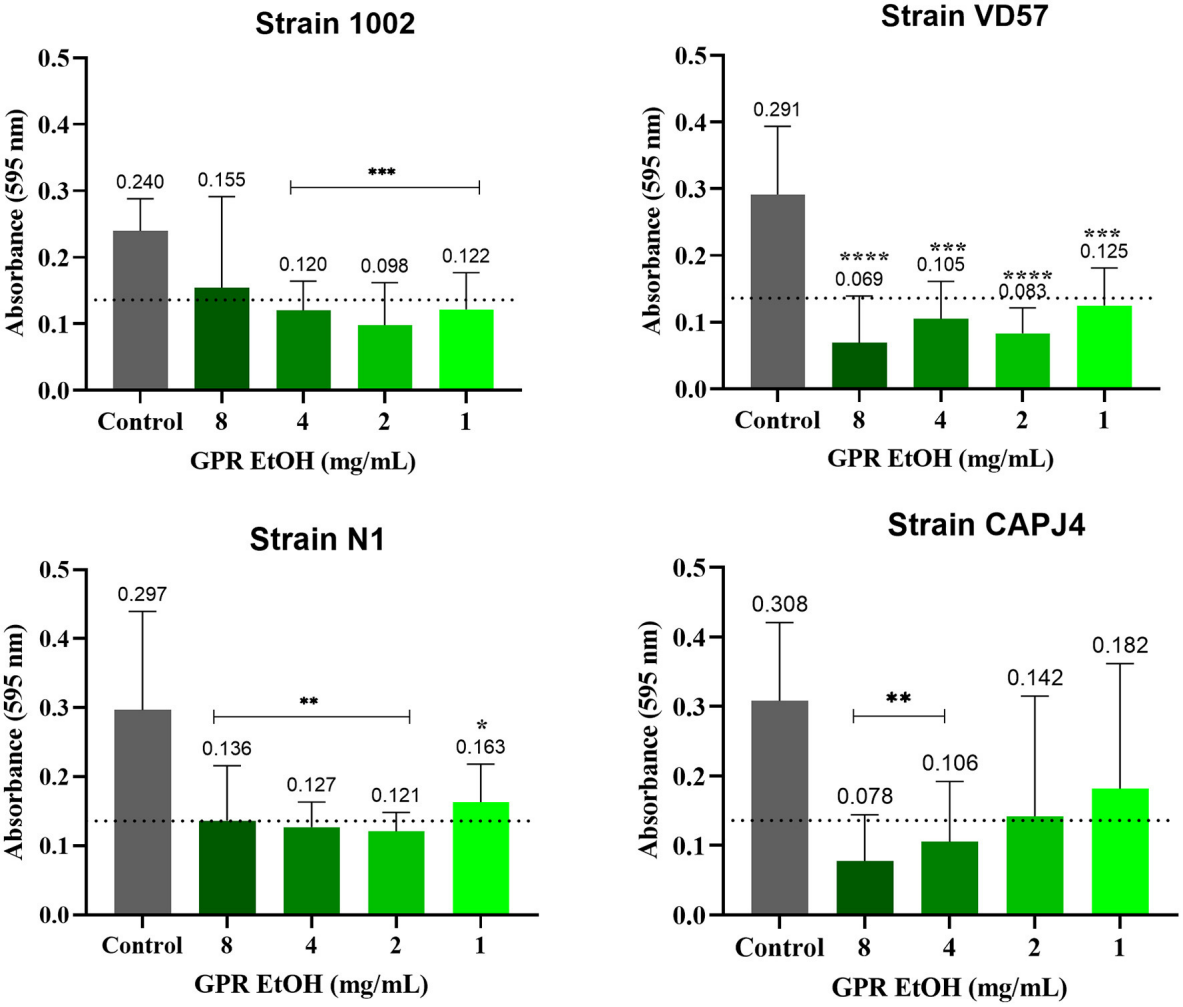
Interference activity of the green propolis ethanolic extract (GPR EtOH) in the formation of *C. pseudotuberculosis* biofilm: Strain 1002, Strain N1, Strain VD57, Strain CAPJ4. The dashed line in the graphs represents the cutoff point for significant biofilm production. Asterisks indicate statistical differences between untreated and treated biofilms analyzed using the one-way ANOVA and *t*-tests. *p < 0.05; **p < 0.04; ***p < 0.003; ****p < 0.0001.

The ethanolic extract of green propolis interfered with the formation of *C. pseudotuberculosis* biofilm. The interference action was more evident on strains VD57 and CAPJ4 with a percentage of interference >80% at a concentration of 8 mg/mL. The N1 strain was the more resistant one (Table 5). After 48 h of incubation, the green propolis ethanolic extract prevented the formation of biofilm among the isolates. It was possible to observe a weak biofilm formation by the VD57 and CAPJ4 strains when treated with the lowest concentrations of green propolis ethanolic extract (Figure 4).

**Table 5:** Interference by the green propolis ethanolic extract in the formation of *C. pseudotuberculosis* biofilm.

***C. pseudotuberculosis* strain**	**Green propolis ethanolic extract concentration (mg/mL)**			
	**8**	**4**	**2**	**1**
1002	53 ± 33	48 ± 19	60 ± 24	48 ± 24
N1	44 ± 30	46 ± 20	45 ± 21	30 ± 18
VD57	80 ± 24	67 ± 17	74 ± 12	61 ± 18
CAPJ4	82 ± 16	75 ± 20	71 ± 31	53 ± 42

*The results are expressed as a percentage of reduction in biofilm formation (%) and correspond to the means and standard deviations of three independent experiments of bacterial cells treated with different concentrations of propolis extract for 48 h*.

The supercritical extract of red propolis prevented the formation of biofilm among most isolates in the highest concentrations tested as did the green ethanolic extract (Table 6). However, a lower interference activity of the supercritical red extract was observed for the 1002 strain at all concentrations.

**Table 6:** Interference by the supercritical extract of red propolis in the formation of *C. pseudotuberculosis* biofilm.

***C. pseudotuberculosis strain***	**Red propolis supercritical extract concentration (mg/mL)**			
	**8**	**4**	**2**	**1**
1002	21 ± 14	36 ± 17	34 ± 31	48 ± 33
N1	49 ± 9	59 ± 10	43 ± 16	46 ± 19
VD57	87 ± 7	80 ± 8	73 ± 3	65 ± 17
CAPJ4	68 ± 8	80 ± 7	82 ± 9	79 ± 17

*The results are expressed as a percentage of reduction in biofilm formation (%) and correspond to the means and standard deviations of three independent experiments of bacterial cells treated with different concentrations of propolis extract for 48 h*.

**Figure 5:**
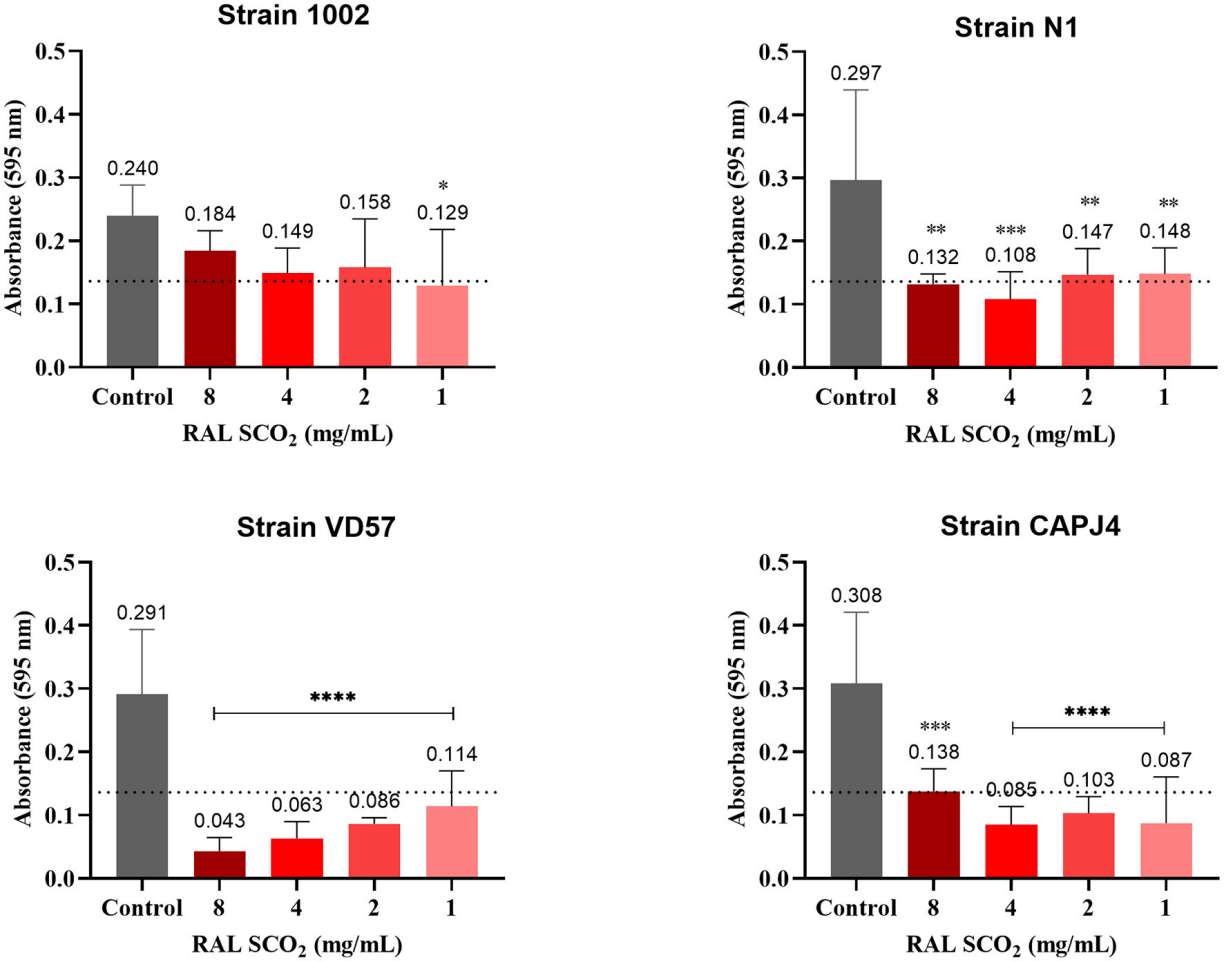
Interference activity of the supercritical extract of red propolis (RAL SCO_2_) in the formation of *C. pseudotuberculosis* biofilm: Strain 1002, Strain N1, Strain VD57, Strain CAPJ4. The dashed line in the graphs represents the cutoff point for significant biofilm production. Asterisks indicate statistical differences between untreated and treated bacterial cells analyzed using one-way ANOVA and *t*-tests. *p < 0.05; **p < 0.04; ***p < 0.003; ****p < 0.0001.

An interference action of the supercritical extract of red propolis in the formation of biofilm was observed among the isolates. The red propolis supercritical extract prevented the initial formation of biofilm by the VD57 and CAPJ4 strains. Strains 1002 and N1 showed a weak biofilm formation after 48 h of incubation with the red propolis supercritical extract (Figure 5).

### Scanning Electron Microscopy

**Figure 6:**
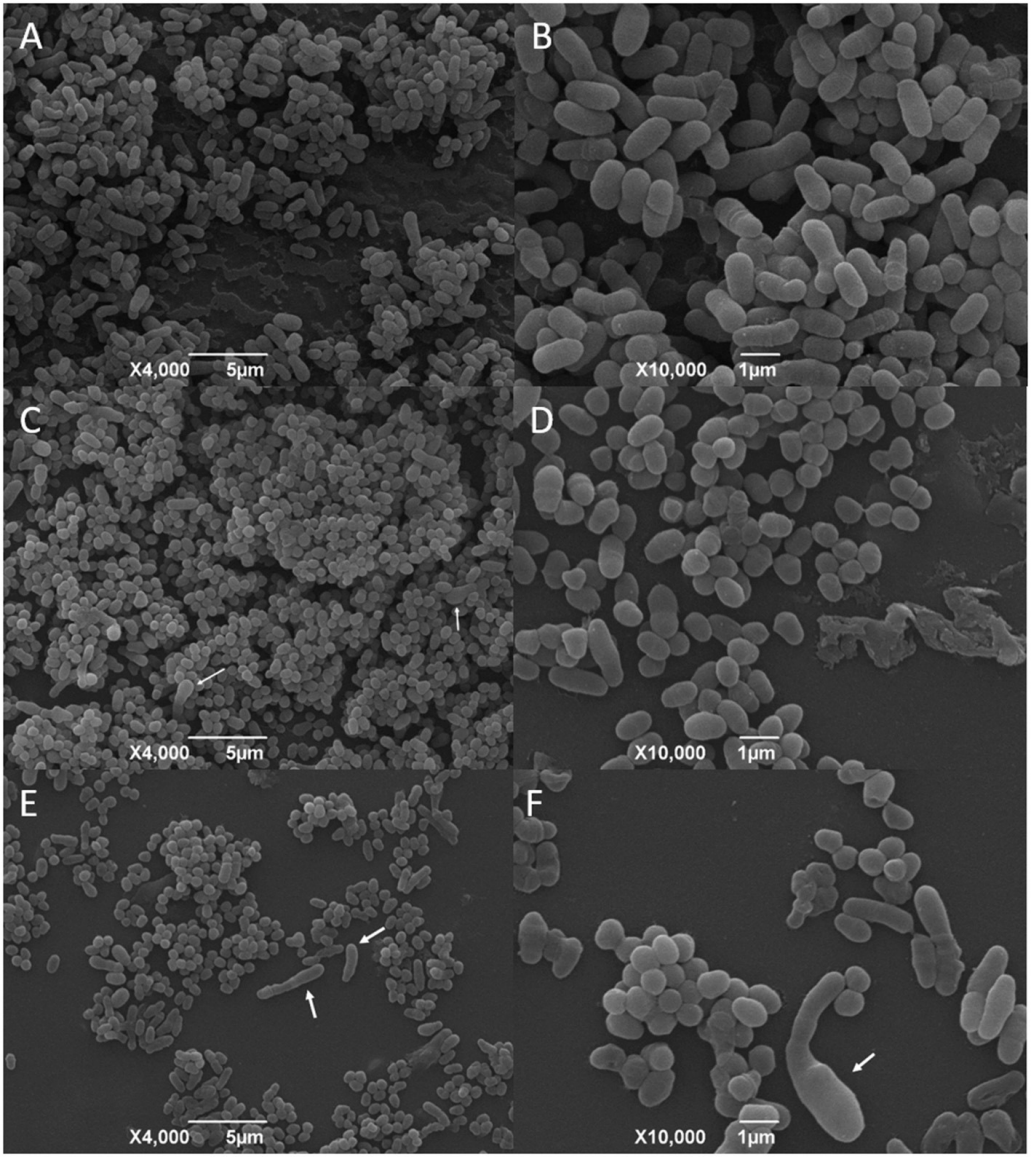
SEM analysis of *C. pseudotuberculosis* biofilm treated or not with ethanolic green propolis extract. **(A,B)** represent the bacterial biofilm without any treatment, and **(C–F)** show the biofilms treated with the green propolis ethanolic extract. The white arrows indicate cellular deformities not seen in the planktonic bacterial cells and in the not-treated biofilm.

**Figure 7:**
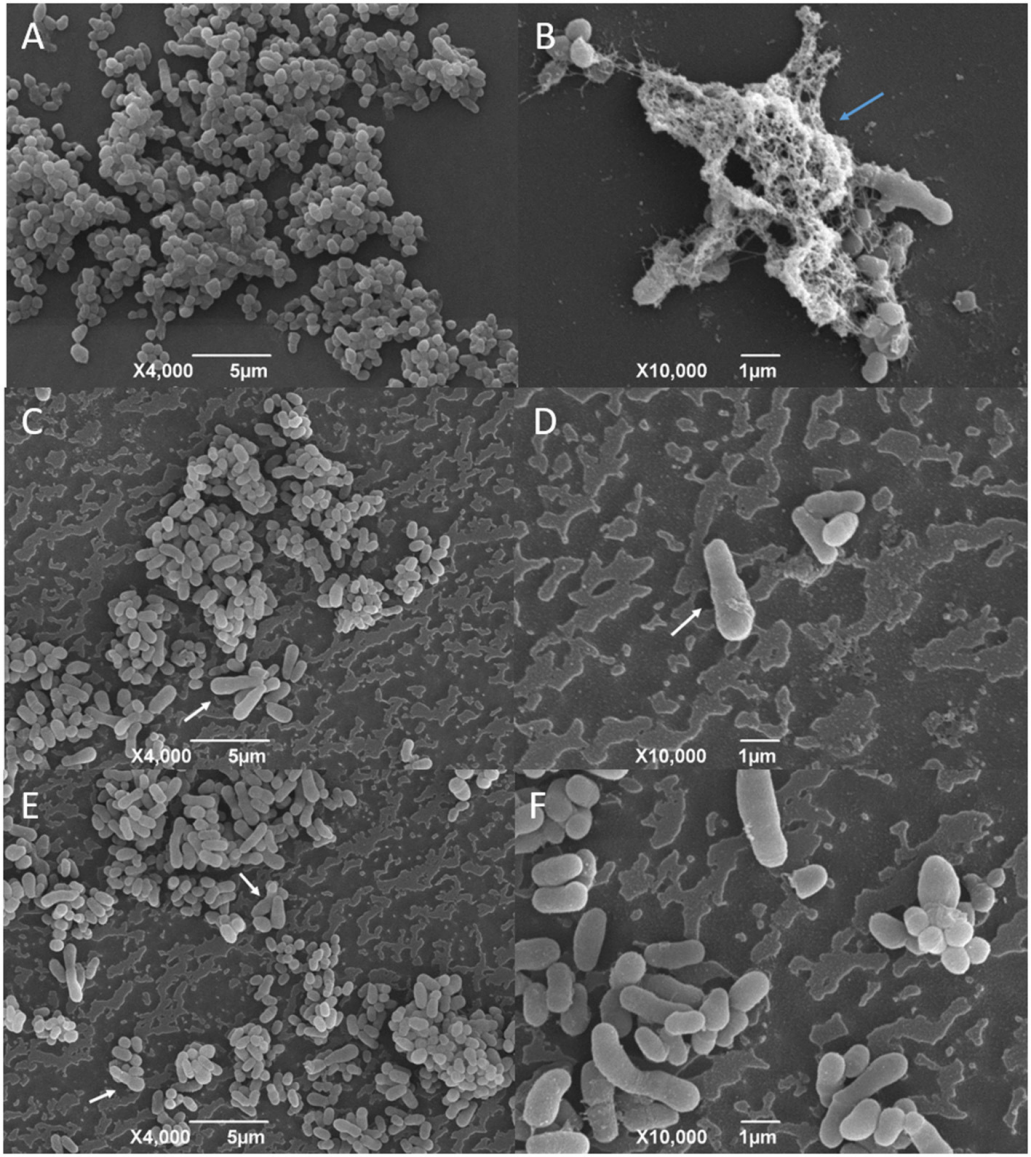
SEM analysis of *C. pseudotuberculosis* biofilm treated or not with supercritical red propolis. **(A)** and **(B)** represent the bacterial biofilm without treatment, and **(C–F)** show the biofilms treated with the red propolis supercritical extract. The blue arrow indicates the presence of an exopolysaccharide matrix in a mature biofilm. The white arrows indicate cellular deformities not seen in the planktonic bacterial cells and in the not-treated biofilm.

The SEM analysis of the *C. pseudotuberculosis* biofilm after a 48-h incubation showed that the bacteria presented a cell morphology like the planktonic cells, showing pleomorphic shapes, such as cocci and rods, and varied sizes (Figures 6A,B and 7A). It was possible to notice the presence of a mature biofilm (Figure 7B), whose main characteristic is the formation of an amorphous exopolysaccharide matrix.

The effects of the green propolis ethanolic and of the supercritical red propolis extracts in the consolidated biofilms are shown in Figures 6, 7, respectively. No significant changes were observed regarding the disruption of the consolidated biofilm of *C. pseudotuberculosis*. The biofilm showed changes in the bacterial morphology with a predominance of cocci forms and the absence of an amorphous extracellular matrix that was once found in untreated biofilms. Other changes in the cell morphology were observed in a few bacterial cells, which showed an increase in size and deformity.

## Discussion

*C. pseudotuberculosis* is an infectious agent of great importance in small ruminant breeding because it causes CLA, leading to significant economic losses related to the reduced productivity and reproductive efficiency in infected animals. The treatment with conventional antibiotics is not effective and is currently restricted to the removal, cleaning, and disinfection of superficial lesions. Thus, we identified in this study that different types of Brazilian propolis extracts can act as antibacterial agents against *C. pseudotuberculosis*, being also able to prevent the formation of biofilm by this bacterium.

The results of the susceptibility tests confirm that the reference strains of *C. pseudotuberculosis* are sensitive to conventional antibiotics (23, 31, 32). Some studies show that different species of *Corynebacterium* may be resistant to antibiotics as a consequence of the presence of genes located on plasmids that confer resistance to streptomycin, erythromycin, chloramphenicol, and tetracycline (33–35) and also to a resistance profile related to the activity beta-lactamases (36). However, our results are similar to the study described by Rhodes et al. (37), who did not find significant phenotypic differences in the antimicrobial resistance profiles of *C. pseudotuberculosis* strains isolated over a period of 16 years (1996–2012).

It is important to consider that surgical treatment is the only treatment currently available for CLA (2, 38), and the lack of use of antibiotic therapy for CLA treatment represents an absence of selective pressure made by antibiotics and, consequently, less resistance development. A selective pressure made by the continuous use of antibiotics characterizes an important precondition for the development of multiresistant strains (39), which most likely is not happening in the breeding of small ruminants and for this specific pathogen. Although the *in vitro* results show excellent action of antimicrobials, the treatment of CLA is refractory *in vivo*, probably due to the thick fibrous capsule around the typical lesions and the thick and caseous nature of the content inside the capsule (4, 40).

It is known that the chemical composition and antimicrobial activity of propolis vary quantitatively throughout the year due to changes in the botanical sources (41). This situation is evident in the Northeast region of Brazil, which presents two distinct seasons (dry and rainy), and this particular issue influences the concentration and composition of metabolites and, consequently, antibacterial activity, which can be translated into a higher or lower MIC (7). Phytochemical evidence based on UV-VIS spectra, RP-HPLC, and GC-MS, shows that *Dalbergia ecastophyllum (L.) Taub*. is the main botanical source of red propolis in Alagoas state (42). Bees of the species *Apis mellifera* collect resinous material from leaf buds of *Baccharis dracunculifolia* (main botanical source) with deposition of green propolis in the hive (43). Brown propolis, found in the South (Paraná and Santa Catarina states) and Midwest (Mato Grosso) regions of Brazil, has varied botanical sources, but it can cite as major sources the flowering of *Luehea* sp. (açoita-cavalo), *Piptadenia falcata* (angico-do-cerrado), *Tabebuia* spp. (ipes), *Tabebuia caraiba* (for-everything), *Vernonia* spp. (assa-fish), and *Cecropia pachystachya* (embaúba) (44–46).

Propolis is the target of several studies because it is a promising source of bioactive compounds, such as phenolic compounds, flavonoids, isoflavonoids, triterpenoids, prenylated benzophenones, caffeic acid, ellagic acid, p-coumaric acid, vitexin, luteolin, rutin, quercetin, and apigenin, which confer mainly antibacterial activities (7). Considering the scarcity of studies involving the use of natural products against *C. pseudotuberculosis*, we evaluated the antibacterial action of the supercritical and ethanolic extracts of propolis and saw that the extracts were effective against the reference strains of this species.

The red propolis extracts obtained by supercritical fluid extraction were highly effective against *C. pseudotuberculosis* with better antibacterial activity than the ethanolic red propolis extract. Different extraction methods can provide extracts with different concentrations of active compounds (polysaccharides, flavones, and terpenes) (47), and it is important to note that the smaller amount of some of these bioactive compounds in the supercritical extract of red propolis (14) did not interfere with the antimicrobial activity, probably due to the action of active compounds contained in this type of propolis, such as isoflavonoids isovestiol, neovestiol, and vestiol, which have already demonstrated antibiotic action against *Staphylococcus aureus, Bacillus* sp., and *Pseudomonas aeruginosa* (48).

When we evaluated the action of green propolis extracts, we realized that the green propolis ethanolic extract was more effective against *C. pseudotuberculosis* strains. The antibacterial action of this type of propolis is due to the large amount of antioxidant compounds and a higher content of phenolic acids, flavonoids, p-coumaric acid, and Artepillin C (14), which, acting in synergism with other components of the extract, are effective in inhibiting bacterial growth of some bacteria, such as methicillin-sensitive (MSA) and methicillin-resistant *S. aureus* strains (MRSA) (27). The antimicrobial potential of green propolis ethanolic extracts against clinical isolates of *C. pseudotuberculosis* was also observed by Kalil et al. (2), and ethanolic green propolis concentrations of 1 and 2 mg/mL inhibited the growth of bacterial isolates by 52%; 48% of the isolates showed MBC at concentrations of 1 and 2 mg/mL, and only six of the isolates were resistant to the action of the extracts.

The brown propolis extract conferred a low antibacterial activity, and this result is related to the lower concentrations of phenols, flavonoids, and antioxidant activity in this type of propolis, culminating in a lower biological potential and, consequently, higher MIC_100_ values when compared with extracts of green and/or red propolis (14). This finding agrees with the study carried out by Gomes et al. (46), who found high MIC values ranging from 2.25 to 18.5 mg/mL for Gram-positive bacteria, and *Staphylococcus* sp. showed an MIC of 9.3 mg/mL, indicating a greater resistance of this genus to the brown propolis extract.

We note that the reference strains of *C. pseudotuberculosis* were able to form biofilm on an abiotic surface. In general, the bacterial characteristics that determine the degree of attachment to such surfaces are of a physical–chemical nature (49), such as temperature, pH, and ionic strength, and most notably hydrophobicity, which is determined by the general composition of the bacterial surface (50). In *C. pseudotuberculosis*, N-acetylmuramoyl-L-alanine amidase and galactose-1-phosphate uridylyltransferase are involved in biofilm formation and exopolysaccharide biosynthesis (24). The extracellular matrix that involves the mature biofilm is composed mainly of glycoproteins, carbohydrates, lipids, and nucleic acids with the carbohydrates providing the basic structure for the biofilms, allowing the stratification of the bacterial population and the exchange of nutrients and waste (51).

The effects on bacterial biofilm of natural products are mainly based on the inhibition of the formation of the exopolymeric matrix, suppression of cell adhesion and fixation, interrupting the generation of extracellular matrix, and a decreased production of virulence factors, thus blocking the quorum-sensing network and the development of the biofilm (52). In our study, we observed a lower efficacy of propolis extracts in *C. pseudotuberculosis* consolidated biofilms, most likely because bacteria in biofilms are inherently more tolerant to antimicrobial treatment when compared directly with planktonic cells of the same strain (53), and concentrations used herein were based on MIC_100_ values obtained for planktonic cells. This result is similar to the finding by Djais et al. (54), in which all concentrations of propolis extracts tested did not disrupt the *Streptococcus mutans* biofilm. Djais et al. (54) attribute that increased *in vitro* production of biofilm can be related to the presence of sucrose, glucan, fructose, and other polysaccharides in the TSB culture medium, which provide the bacteria with enough substrate to initiate the formation of dental biofilms; thus, the *in vitro C pseudotuberculosis* greater resistance to the extracts can be also linked to factors related to bacterial culture in TSB broth.

We also observed a greater sensitivity of the consolidated biofilm of the CAPJ4 virulent strain against the ethanol extract of green propolis. Considering a possible correlation between the virulence of several strains of mycobacteria and their susceptibility to the ethanol extract of propolis, Scheller et al. (55) did not find a complete correlation as few strains that were resistant to the ethanol extract of propolis were nonvirulent. Sá et al. (24) observed that CAPJ4 differentially synthesizes penicillin-binding protein, which participates in peptidoglycan formation and exhibits upregulation of N-acetylmuramoyl-L-alanine amidase and galactose-1-phosphate uridylyltransferase, which are involved in biofilm formation and exopolysaccharide biosynthesis. Yaacob et al. (51) believe that changes in Raman spectral characteristics are useful to explain the heterogeneity of the different stages of the *C. pseudotuberculosis* biofilm as well as the different antibiotic sensitivity profiles. A metabolomic analysis demonstrated that biofilm formation in *Helicobacter pylori* may be influenced by its lipidome, suggesting that there may be a difference in the membrane composition of strains with high and low biofilm formation (56). Furthermore, the lipid composition of bacterial cells also shows an impact on the susceptibility to antimicrobials, probably due to alterations in the penetration of the membrane to antimicrobials (57). As far as we know, besides the fact that the natural compounds can act on different biofilm formation processes developed by the bacterium, there has been no study conducted until now about the molecular pathways that are involved with the biofilm formation in *C. pseudotuberculosis*, and little can be speculated about this particular aspect.

In contrast to our results, several studies show high antibiofilm activity of propolis extracts against Gram-positive and Gram-negative bacteria. De Marco et al. (58) conclude that the extracts of propolis and the *Populos nigra* resin are able to inhibit the formation of *P. aeruginosa* biofilm. The ethanolic extracts of propolis tested by Grecka et al. (59) effectively eliminated the biofilm of *S. aureus* as well as planktonic cells with biofilm eradication concentration values (MBEC_50_) of up to 128 μg/mL. The low activity of propolis extracts in the consolidated biofilm of *C. pseudotuberculosis* may be associated with two factors. First, is the direct action on the microorganism, inferring its effect on the permeability of the cell membrane and disruption of the cell wall. This is evident because the antibacterial activity of propolis is greater in Gram-positive than in Gram-negative bacteria due to the species-specific structure of the outer membrane of Gram-negative bacteria (60, 61). Most species of the genus *Corynebacterium* are characterized by a complex architecture of the cell wall, whose outer layer of mycolic acids is functionally equivalent to the outer membrane of Gram-negative bacteria, and in the upper layer, an external surface material composed of polysaccharides, glycolipids, and free proteins, including S-layer proteins, pili, and other proteins can be found (62). The second factor is attributed to the extracellular matrix of the biofilm. The exopolymeric matrix of biofilm-forming species has an innate ability to prevent the penetration of antibacterial agents (63).

In any case, our results are promising because there is recent progress in the research on natural products with the objective to face resistance and tolerance to antibiotics, which are current global problems (42). This becomes more evident when recent studies, as described by Rampacci et al. (64), experimentally demonstrate that increasing MIC concentrations (10x) of azithromycin and rifampicin alone and combined did not eradicate the preformed biofilm of *Rhodococcus equi* although a rifampicin-resistant isolate produced an exceptionally abundant extracellular matrix. Regarding *Corynebacterium diphtheriae* biofilm, all tested strains showed increased biofilm formation over a glass support after treatment with sub MIC concentrations of erythromycin. These results indicate that the biofilm formation resistance induced by these antibiotics may contribute to the failure of antimicrobial therapy against infections by *C. diphtheriae* (65), which differs from our data in which the subinhibitory concentrations of propolis extracts show some efficacy against consolidated biofilm samples of *C. pseudotuberculosis*.

Propolis extracts were more efficient in preventing colonization and reducing the fixation of *C. pseudotuberculosis* on the surface of the microplate than in disrupting a consolidated biofilm. These results indicate that propolis extracts may be useful in biofilm formation intervention strategies, which have been adopted when there is inherent resistance from consolidated biofilms to antimicrobial agents (66, 67). In fact, several studies with propolis extracts from different locations reveal the ability that propolis extracts have in interfering with the formation of bacterial biofilm. The Brazilian green propolis extract can inhibit the growth of *Streptococcus mutans* dental biofilm (68), and the ethanolic extract of Albanian propolis significantly reduces (81%) the early formation of *Pseudomonas aeruginosa* biofilm (69).

We investigated the activity of the green propolis ethanolic extract and of the red propolis supercritical extract against strains of *C. pseudotuberculosis* protected by a mature biofilm. The micrographs obtained through the SEM corroborate the findings obtained by the microplate quantification test and reveal the biofilm formation capacity of the bacterial isolates. Besides the fact that it was not possible to observe changes in the morphology of the biofilm itself, it was possible to observe that the bacterial cells at the propolis-treated biofilm presented changes on its morphology, such as a high frequency of elongated forms, that could not be seen at the propolis not-treated biofilms for the biofilms exposed to both the ethanolic green propolis or the supercritical red propolis extracts. Also, it could not be seen at the treated biofilms the exopolysaccharide matrix that was evident in the not-treated biofilms. According to SEM analysis made by other authors, the survival of bacteria in biofilm can be affected by different treatments with propolis. When an *S. epidermidis* biofilm was treated with ethanolic and ethyl acetate extracts of propolis, the decrease in survival was only 10%. On the other hand, the action of chitosan-propolis nanoparticles reduced survival by 70%, resulting in a breakdown of the biofilm and a decrease in the number of bacteria (49). Bryan et al. (70) observed that the biofilm of *S. aureus* was more susceptible to the ethanolic extract of Russian propolis (3% w/v) when compared to the *E. coli* biofilm, and only the treatment with propolis at 20% w/v resulted in a complete inactivation of the bacterial biofilms, indicating that the antibiofilm activity is dose-dependent. In such a way, we can conclude that the maximum concentration used in our study must be considered as suboptimal due to the survival and alterations found in the biofilms of *C. pseudotuberculosis* even without a significant disruption.

The strains of *C. pseudotuberculosis* were sensitive to antibiotics as well as to ethanolic and supercritical propolis extracts. The antimicrobial potential of propolis extracts has been described against antibiotic-sensitive clinical isolates of *Streptococcus pyogenes*, clinical isolates of *Candida* sensitive to antifungals, and MRSA (71). When in synergism with antibiotics that inhibit bacterial cell wall synthesis (vancomycin and oxacillin), propolis extracts inhibited the growth of *S. pyogenes*, MRSA, and vancomycin-resistant *Enterococcus* (VRE) by more than 3.5 log_10_ (72). In general, propolis bioactive compounds positively modulate the antimicrobial resistance of multiresistant bacteria, increasing the spectrum against different types of microorganisms (73), including antibiotic-resistant biofilm-forming bacteria (70). It must be considered that, besides the fact that these strains were susceptible to clinically relevant antibiotics, these compounds are not able to penetrate the granuloma membrane and, in this way, do not represent an effective treatment against CLA. The use of antimicrobials can be directed to postsurgical treatment when these compounds can be used to prevent the dissemination of the bacteria and reactivation of the disease after the removal of the lesions, and in this way, propolis can be addressed as an interesting treatment because it would be able not only to prevent the reincidence of the disease, but can also prevent environmental contamination, avoid antibiotic selective pressure, accelerate the surgical wound-healing process, and be a feasible and low cost treatment.

In conclusion, we were able to verify that *C. pseudotuberculosis* strains maintain a high sensitivity to conventional antibiotics. The response of the *C. pseudotuberculosis* planktonic isolates varied between the ethanolic and supercritical extracts of green, red, and brown propolis. The supercritical extract of red propolis and the ethanolic extract of green propolis showed the highest antibacterial activity among the isolates, being able to interfere in the formation of biofilm. However, the action of propolis extracts in a consolidated and structured bacterial community was not significant.

## Data Availability Statement

The original contributions presented in the study are included in the article/Supplementary Material, further inquiries can be directed to the corresponding author.

## Author Contributions

LS, DR, and MK performed the experiments. MU-G and BM obtained the propolis extracts. LS, NS, and RP wrote the manuscript and performed the data analysis. VA, RM, and RP designed the experiments. VA, RM, and RP critically reviewed the manuscript. All authors contributed to manuscript revision, read, and approved the submitted version.

## Conflict of Interest

The authors declare that the research was conducted in the absence of any commercial or financial relationships that could be construed as a potential conflict of interest.

## Publisher's Note

All claims expressed in this article are solely those of the authors and do not necessarily represent those of their affiliated organizations, or those of the publisher, the editors and the reviewers. Any product that may be evaluated in this article, or claim that may be made by its manufacturer, is not guaranteed or endorsed by the publisher.
