# Supplementary material for: Activity of Ethanolic and Supercritical Propolis Extracts in Corynebacterium pseudotuberculosis and Its Associated Biofilm
Source: Front Vet Sci. 2021 Sep 1;8:700030. doi: 10.3389/fvets.2021.700030 (PMC8440938; doi:10.3389/fvets.2021.700030)
Supplement: Supplementary file 1 [file Table_1.pdf]

Supplementary Table 1. Content of humidity, total solids, total ash, raw protein, total lipids, raw fiber, minerals and determination of water activity of propolis samples from different regions of Brazil. RAL – red propolis from Alagoas State; GRP – green propolis from Paraná State; BSC – brown propolis from Santa Catarina State. These parameters were defined by Machado et al (14).

**Parameters**	**Propolis types**		
	**RAL**	**GRP**	**BSC**
**Humidity (%)**	7.03 ± 0.42	7.13 ± 0.12	7.07 ± 0.10
**Total solids (%)**	92.97 ± 0.42	92.87 ± 0.12	92.93 ± 0.10
**Total ash (%)**	0.96 ± 0.03	3.15 ± 0.03	1.73 ± 0.19
**Protein (%)**	2.30 ± 0.05	9.98 ± 0.83	3.90 ± 0.49
**Lipids (%)**	66.33 ± 0.01	48.72 ± 1.29	74.31 ± 5.69
**Fiber (%)**	7.66 ± 0.90	20.89 ± 1.39	7.29 ± 0.30
**Aw (%)**	0.689 ± 0.01	0.688 ± 0.02	0.657 ± 0.02
**Na (mg/Kg)**	10.10 ± 0.41	3.00 ± 0.01	15.30 ± 1.05
**K (mg/Kg)**	28.70 ±3.16	331.70 ± 15.81	110.3 0± 6.77
**Li (mg/Kg)**	4.50 ± 0.64	1.80 ± 0.01	3.10 ± 1.00
**Ca (mg/Kg)**	40.10 ± 0.72	9.60 ± 0.01	5.90 ± 0.04
