# Supplementary material for: Activity of Ethanolic and Supercritical Propolis Extracts in Corynebacterium pseudotuberculosis and Its Associated Biofilm
Source: Front Vet Sci. 2021 Sep 1;8:700030. doi: 10.3389/fvets.2021.700030 (PMC8440938; doi:10.3389/fvets.2021.700030)
Supplement: Supplementary file 2 [file Table_2.pdf]

Supplementary Table 2. Content of total phenolic compounds, flavonoids, antioxidant activity by the DPPH method (IC_{50}), and determination of radical scavenging activity by the ABTS method (%) of the extracts of different samples from Brazilian propolis. The extracts were obtained by the conventional ethanolic (EtOH) and supercritical (SCO_2) extraction methods. RAL – red propolis from Alagoas State; GRP – green propolis from Paraná State; BSC – brown propolis from Santa Catarina State. These parameters were defined by Machado et al (14).

Propolis extracts	Phenolic compounds (mg EAG/g)	Flavonoids (mg EQ/g)	DPPH (IC_{50})	ABTS (%) (Trolox 1 mg/mL)
**RAL SCO_2**	157.16 ± 0.01	40.65 ± 0.01	183.11 ± 0.31	82.80 ± 3.50
**RAL EtOH**	198.77 ± 0.01	58.19 ± 0.01	44.29 ± 0.29	98.20 ± 1.30
**GRP SCO_2**	118.14 ± 0.03	29.71 ± 0.01	85.34 ± 0.23	73.80 ± 1.80
**GPR EtOH**	179.52 ± 0.01	39.90 ± 0.01	157.39 ± 0.26	89.90 ± 1.80
**BSC SCO_2**	218.09 ± 0.01	31.38 ± 0.01	331.88 ± 0.09	72.70 ± 5.30
**BSC EtOH**	117.03 ± 0.01	27.97 ± 0.01	163.00 ± 0.31	89.80 ± 1.20
